# A comparison of panoramic views reformatted from CBCT scans using manual or automated workflows: an evaluation of geometric accuracy and subjective image quality

**DOI:** 10.1007/s00784-026-07086-1

**Published:** 2026-08-25

**Authors:** Ali Fahd, Mohamed T. Ellabban, Michael M. Bornstein, Ola M. Rehan

**Affiliations:** 1 Oral and Maxillofacial Radiology, Department of Clinical Dental Sciences, Faculty of Dentistry, Ibn Sina University for Medical Sciences, Amman, Jordan; 2 Department of Orthodontics and Dentofacial Orthopedics, Faculty of Dentistry, Assiut University, Assiut, Egypt; 3 Department of Oral Health & Medicine, University Center for Dental Medicine Basel UZB, University of Basel, Basel, Switzerland; 4 Department of Oral and Maxillofacial Radiology, Faculty of Dentistry, Cairo University, Giza, Egypt

**Keywords:** Cone Beam Computed Tomography, Reformatted panorama, Linear measurements, Subjective image quality, Plane orientation, Curved planar reformatting

## Abstract

**Objectives:**

This study aimed to compare (1) the geometric accuracy and (2) subjective image quality of CBCT reformatted panoramic images generated using three reconstruction workflows: automatic reconstruction, nasal spine plane-oriented manual reconstruction, and occlusal plane-oriented manual reconstruction.

**Materials and methods:**

Twenty-eight CBCT datasets were included. Three panoramic reconstruction protocols were assessed. Linear measurements were obtained on axial images (as a reference) and on the corresponding reformatted panoramic images. Measurements included (for geometric accuracy): mesiodistal crown width of a selected maxillary central incisor; inter-radicular distance at the alveolar crest between adjacent anterior teeth; and panoramic interslice distance (PISD), defined as the distance between the primary panoramic line (center of the panoramic curve) and the secondary panoramic line at the measurement site. A 7-item binary visual scoring system was used to compare subjective image quality across protocols.

**Results:**

Crown-level measurements differed significantly between axial images and reformatted panoramic images across all protocols. Bone-level measurements showed significant differences in G1 and G3, while there was no significant difference in the G2 protocol. In all groups, measurement error (absolute and percentage) showed a significant positive correlation with PISD. Subjective image quality scores differed across protocols.

**Conclusions:**

CBCT-reformatted panorama demonstrated protocol-dependent differences for linear measurements and subjective image quality. Measurement errors increased as the measurement sites deviated from the center of the drawn panoramic curve.

**Clinical Relevance:**

Reformatted panorama requires cautious interpretation and should not be relied upon for precise quantitative assessments. Cross-verification with other views is essential before definitive clinical decisions.

## Introduction

Oral and maxillofacial radiology comprises various imaging techniques that are frequently used in general dental practice [1–3]. In addition to radiographic interpretation, dental imaging is also used for patient communication and increasingly for educational purposes supported by digital workflows [1, 3]. Panoramic views are two-dimensional dental imaging with a general overview of the jaws used for basic diagnostics in various fields such as orthodontics and oral surgery [4]. Cone Beam Computed Tomography (CBCT) is a more recent and widely recognized three-dimensional (3D) dental imaging modality. It is used across various clinical fields such as implant dentistry, orthodontics, periodontics, and many other specialties for diagnosis and treatment planning [1, 3, 5, 6].

One feature of CBCT scans is that they allow the reconstruction of panorama-like views that could overcome the inherent limitations of classic panoramic views, which include geometric and visual inaccuracies related to imaging physics and errors in patient positioning [7]. Generally, the curved and wide-view nature of dental panoramic imaging causes dimensional discrepancies with visual and geometric distortions that are also affected by patient orientation (head position) and the anatomical location of the measurement [8]. Published literature assessing the reliability of linear measurements on panoramic views reformatted from CBCT scans remains limited [9]. In addition, there is no universally accepted protocol for panoramic image reconstruction, such as reference plane orientation, axial level selection, curve design and thickness, control point placement, display settings, and the level of automation provided by the software [3, 10].

This uncertainty creates practical challenges and risks for clinicians who use CBCT reformatted panoramic images for both quantitative measurements and qualitative interpretation. Most dental CBCT software allow measurements in reformatted panoramic views, and many clinicians find this easier to use in their workflows due to familiar orientation and simplicity. Accordingly, this study aimed to compare (1) the geometric accuracy and (2) subjective image quality of CBCT reformatted panoramic images generated using one automatic and two standardized manual reconstruction protocols, and to investigate the relationship between measurement error and deviation from the center of the panoramic curve.

## Materials and methods

This retrospective radiographic study was approved by the institutional ethical review board (Cairo University, Faculty of Dentistry, Research Ethics Committee, Code: 29-6-24). All CBCT datasets were acquired using a Planmeca ProMax 3D unit (Planmeca Oy, Helsinki, Finland) with standard 360° exposure at 90 kV, 6 mA, 0.200 mm voxel size, and a field of view of 11 × 8 cm or larger and were processed using the device-inherent Romexis software (version 5.4; Planmeca Oy, Helsinki, Finland).

### Eligibility criteria and sampling

CBCT volumes were eligible if the field of view included both dental arches and extended posteriorly to include at least part of the mandibular ramus. Included cases had bilateral first molars in occlusion (to be used in volume orientation), intact maxillary incisors without restorations (to standardize crown-level measurement), and images free of motion and other artifacts that would compromise reconstructions or linear measurements.

### Sample size

Sample size estimation was performed using G*Power (version 3.1.9.2; Heinrich-Heine-Universität Düsseldorf, Düsseldorf, Germany) [11] based on pilot data (effect size 0.62), with an alpha error set at 0.05 and power at 0.80. A target sample size of 27 datasets was indicated. To account for exclusions, 30 CBCT patient cases were randomly selected, anonymized, and each one was exported as a multi-frame DICOM file. Two cases were excluded (one due to motion artifact and one due to failure of automatic panoramic view reconstruction), yielding a total of 28 datasets for analysis. For each dataset, one maxillary central incisor was selected for linear measurements using a predefined randomization procedure.

### Image preparation and standardization (Fig. 1)

All datasets were prepared under standardized display conditions. Multiplanar reformatted images (axial/coronal/sagittal) and 3D rendering were used to support consistent orientation and measurement level selection. Image display parameters were standardized for viewing consistency (fixing thickness/contrast/density/sharpness at 0.2/0/2000/10, respectively). The 3D rendering was enhanced using the same protocol provided by Fahd et al. [3] to be used as a visual aid to confirm anatomical alignment, standardization, and measurement level consistency across different protocols.

**Fig. 1:**
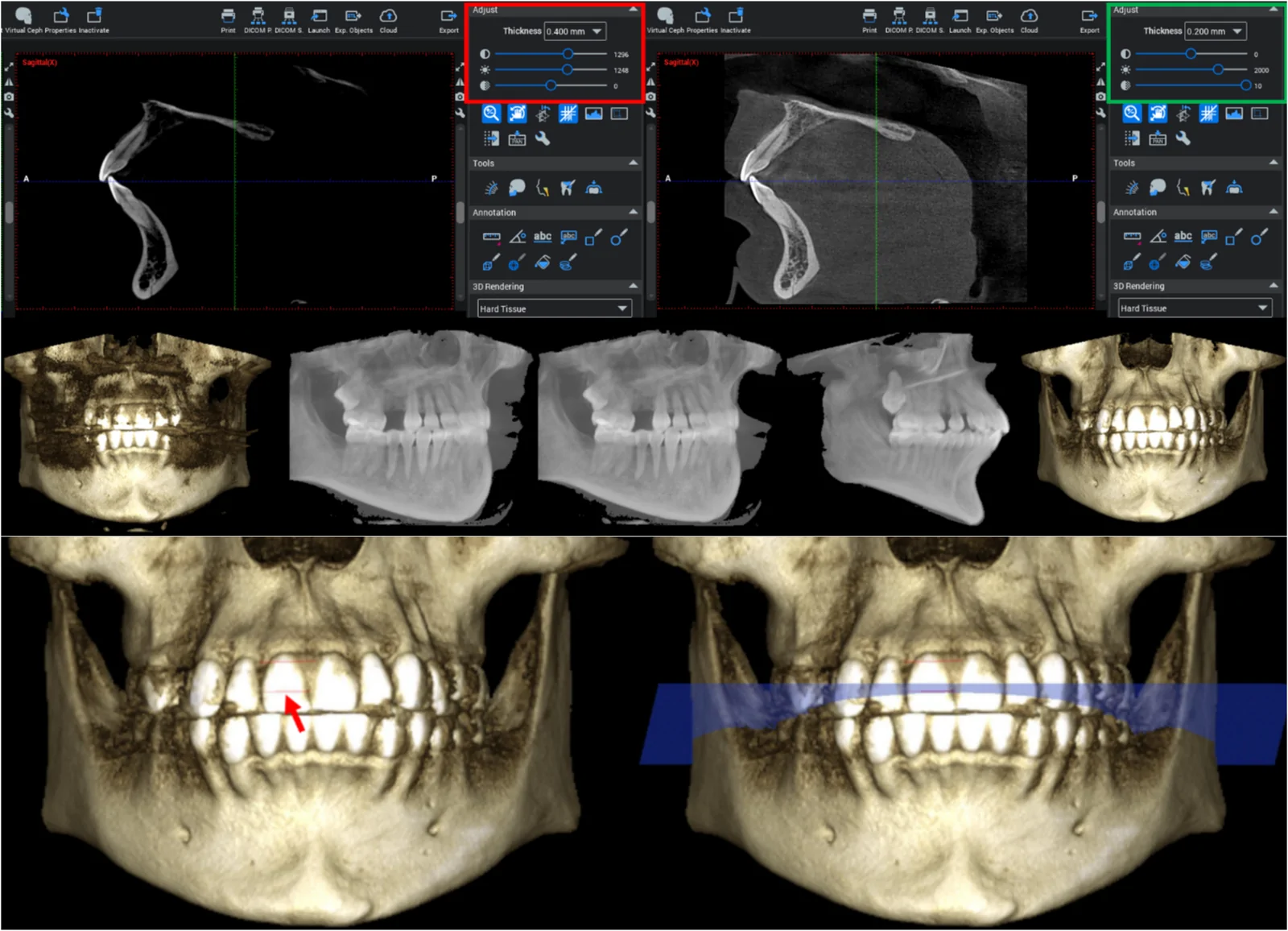
Image preparation and standardization: Comparison of image display settings before and after image enhancement using Romexis software’s display adjustment tools (red and green boxes). Note the original hard palate orientation. The middle row shows the 3D volume rendering before (left) and after enhancement (right), illustrating the workflow. The rendering preset was adjusted to a black-and-white style with higher transparency and lower contrast, enabling clearer identification and removal of noise from multiple viewing angles. Note how the teeth and alveolar crest are clearly visible, so measurement overlays are displayed on the previously enhanced 3D volume rendering to standardize the measurement level across groups. The red measurement line on the crown of the right central incisor (pointed to by the red arrow) indicates the reference level to which the axial plane was aligned each time before measurements

### Reconstruction protocols (groups)

Three reconstruction protocols were evaluated, representing a commonly used fully automatic workflow and two standardized manual reorientation and reformatting workflows:

#### Group 1 (G1 or Protocol 1)

**Fig. 2:**
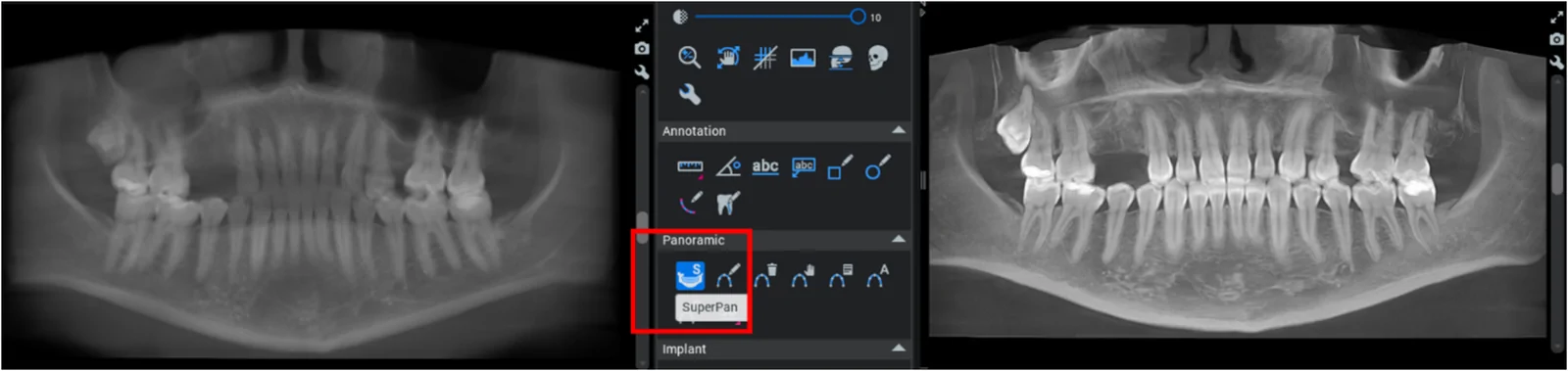
Automatic panoramic reconstruction using the SuperPan tool. The reformatted panoramic image is shown before (left) and after (right) applying SuperPan

Automatic reconstruction. Reformatted panoramic images were generated using the software’s automatic panoramic reconstruction function (SuperPan) (Fig. 2). The software automatically detected the dental arch, generated a panoramic curve, selected the reconstruction thickness, and applied vendor-defined enhancement steps. Because the algorithm operates as a “black box” to the user, the automatically selected panoramic thickness was recorded by linear measurement using the ruler to be used later.

#### Group 2 (G2 or Protocol 2)

**Fig. 3:**
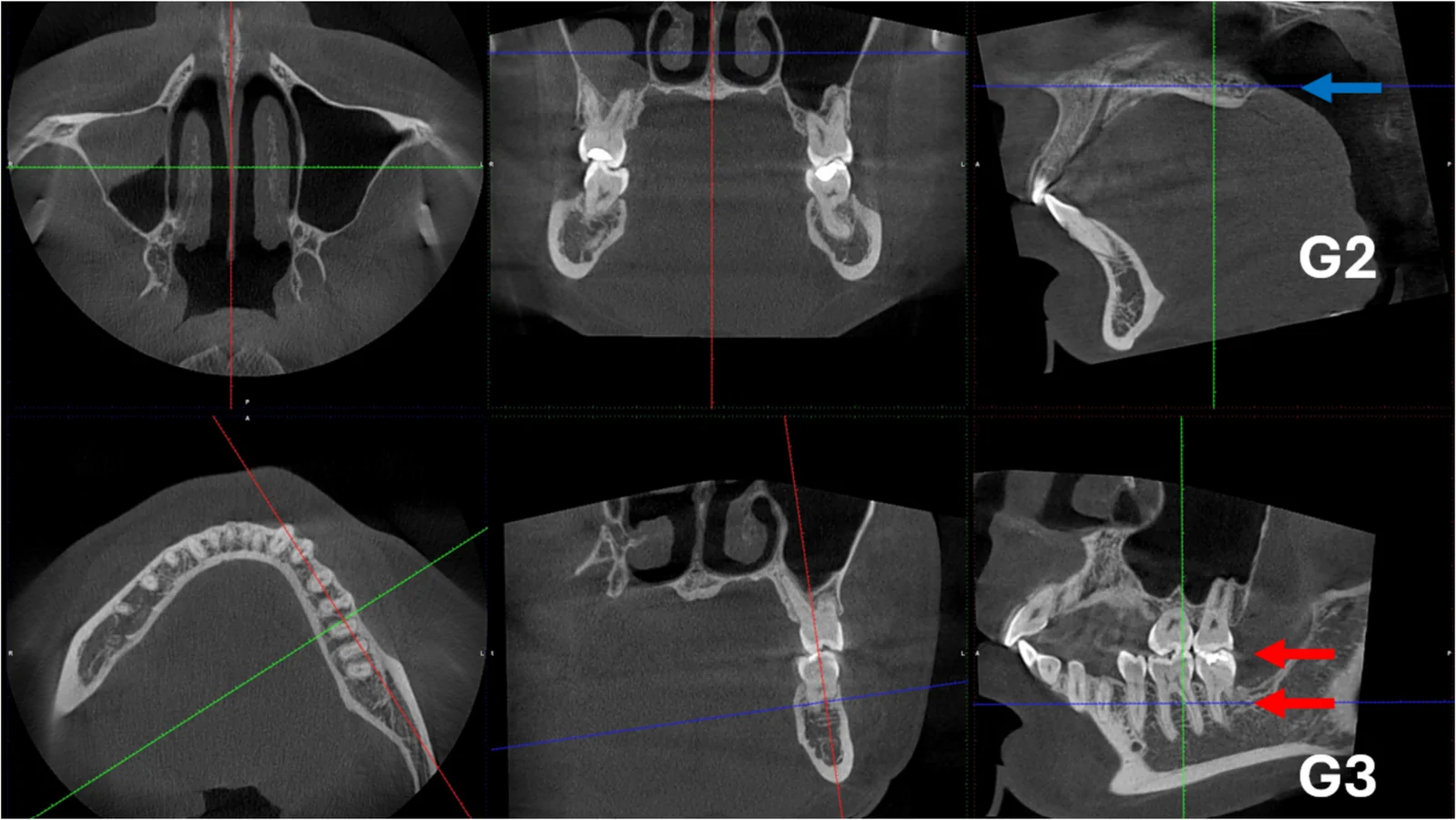
Manual reorientation of the CBCT volume for Group 2 (upper row) and Group 3 (lower row). Orientation was standardized by rotating the volume in the sagittal, coronal, and axial views to align the reference planes for each protocol. Note the final orientation for each protocol where the hard palate is in match with the axial reference line in G2 (blue arrow) and the occlusal plane is in match with the axial reference line in G3 (red arrows)

Nasal spine plane-oriented manual reconstruction. The CBCT volume was reoriented so that the axial reference plane followed the anterior nasal spine–posterior nasal spine line (hard palate plane) as shown in (Fig. 3) (original hard palate orientation is shown in Fig. 1). Orientation was done on the explorer module using reference lines and volume rotation controls; the final orientation was transferred to other modules via the Export View function. A manual panoramic curve was then defined on the axial view at the level of the maxillary anterior alveolar crest using a standardized number of control points (eight points), extending posteriorly to include the visible ramus region. Panoramic curve thickness was fixed at 25 mm (maximum available), selected to minimize exclusion of dental structures in cases with arch width variation while maintaining a consistent parameter across datasets.

#### Group 3 (G3 or Protocol 3)

Occlusal plane-oriented manual reconstruction. The CBCT volume was reoriented so that the occlusal plane was parallel to the axial reference plane (Fig. 3). Panoramic curve definition followed the same standardized approach as in G2 (eight control points, similar posterior extent, and 25 mm panoramic curve thickness).

## Measurements

**Fig. 4:**
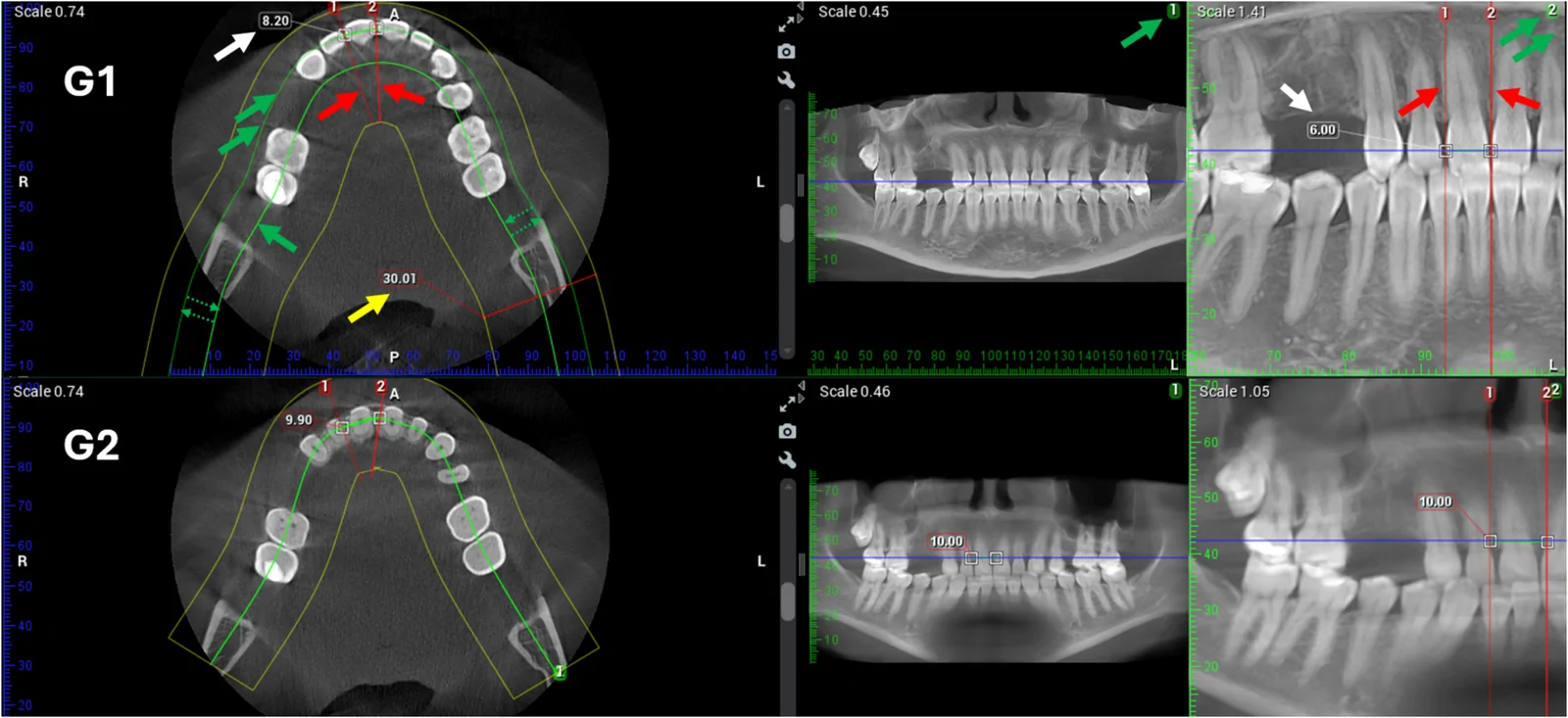
G1: Crown-level measurements on the axial view shown on the left (white arrow) and the corresponding reformatted panorama shown on the right (white arrow). Measurement endpoints were adjusted using the fine-tuning handles (end boxes). The red measurement line, measuring 30.01 mm, pointed to by the yellow arrow, indicates the automatically done panoramic contour thickness, which is the distance between the outer and inner curved yellow lines marking the lingual and labial limits of the reconstruction thickness. Note the difference in thickness between G1 and G2. One of the two green curved lines indicates the center of the panoramic curve, where panoramic curve points are drawn either manually or automatically (single green arrow in axial), and is referred to as panoramic slice 1 (single green arrow in panorama). The secondary curved green line (two green arrows in axial) passes through the measurement site (labial to the centerline) and is referred to as panoramic slice 2 (two green arrows in panorama). The distance between the two green curved lines is the panoramic interslice distance or PISD (dotted green arrows). The two red arrows refer to the reference lines used to accurately place measurement points at the intersection of two lines. Note that the red reference lines are converging palatally in the axial slice. Measurements are 8.2 mm on the axial and 6 mm on the reformatted panorama for the same anatomic location (more than 2 mm difference). G2: Bone-level measurements shown on the axial CBCT image and the corresponding reformatted panoramic image. The primary panoramic line and the secondary line coincide (shown as one line, unlike the upper row, which is G1), indicating alignment or matching of the measurement plane with the center of the panoramic curve. Note the false bone defect visible in the lower arch at the mental region. Measurements are 9.9 mm on axial and 10 mm on panorama (nearly identical)

In each protocol, linear measurements were obtained on the axial image slice (as a reference, derived directly from the volumetric dataset without curved planar synthesis) and the corresponding reformatted panoramic image. Measurements were performed at two levels. The crown level, which was defined as the slice/level showing the maximum mesiodistal dimension of the selected maxillary central incisor. The second level was at the alveolar crest level (bone), defined as the interproximal line between the lateral and central incisors adjacent to the selected central incisor (Fig. 4).

For each group, three measures were recorded:

1. Mesio-distal crown width: maximum mesiodistal width of the selected maxillary central incisor measured on axial and reformatted panoramic images in millimeters (mm).
2. Inter-radicular distance at alveolar crest: distance between the lateral and central incisors adjacent to the selected central incisor at the alveolar crest level, using standardized anatomic boundaries for point placement in mm.
3. Panoramic interslice distance (PISD) in the axial slice: the labio-lingual distance between the primary panoramic line (center of the panoramic curve) and the secondary panoramic line passing through the measurement site in mm (distance of the area of measurement from the center of the panoramic curve in axial view). PISD was recorded to quantify how far the measurement location deviated from the curve center and was hypothesized to relate to measurement distortion.

### Visual scoring system

A 7-item binary (0/1) visual scoring system was used to evaluate panoramic image qualitative appearance and landmark visibility. For each item, a score of “1” indicated that the criterion was clearly met and clinically interpretable, whereas “0” indicated that the feature was not reliably visible or was compromised.

The proposed visual assessment criteria were:

1. Smile line normality: The smile line was defined as the curve formed by the occlusal plane across the dentition, which is normally slightly upwardly curved. A score of 1 was assigned if the smile line appeared normal.
2. DEJ visibility: Dentino-enamel junction (DEJ) visibility was evaluated. A score of 1 was assigned if the DEJ was clearly identifiable.
3. Absence of tooth distortion: Tooth shape was assessed for distortion. A score of 1 was assigned if no clinically relevant alteration in the normal tooth shape was observed.
4. Complete image extension: Horizontal coverage of the image was assessed. A score of 1 was assigned if the image demonstrated the full horizontal field of view.
5. Adequate bone display: Bone coverage was assessed for cutoffs. A score of 1 was assigned if the bony structures were displayed without false defects.
6. Inferior alveolar canal (IAC): A score of 1 was assigned if the IAC was clearly identifiable.
7. Absence of teeth overlap: Overlap between teeth that are normally separated (through axial evaluation) was assessed. A score of 1 was assigned if no such overlap was present.

### Measurements by observers

Before measurements, two observers participated in a calibration session using representative cases not included in the final study sample. The calibration process was designed to standardize volume orientation, panoramic curve construction, measurement point identification, and visual scoring criteria. Following calibration, the two observers independently performed all reconstructions, measurements, and visual scoring. The two observers were oral and maxillofacial radiologists (OMFR) with more than 15 years of experience. Each observer repeated all measurements twice to allow for assessment of intra-observer repeatability and inter-observer agreement. The case order was randomized, and the assessors were blinded to group identity during measurement extraction (another individual not involved in measurement).

### Statistical analysis

Statistical analyses were performed using IBM SPSS Statistics (version 22). Normality was assessed using the Shapiro–Wilk test. Within each group, paired t-tests compared axial versus reformatted panoramic measurements. Between-group differences in measurement discrepancies were assessed using one-way ANOVA with Tukey post-hoc testing (alpha = 0.05). Pearson correlation assessed the relationship between PISD and measurement error (absolute and percentage). Visual scores were compared using the Kruskal–Wallis test followed by Dunn’s post-hoc comparisons with Bonferroni adjustment. For continuous measurements, intra- and inter-observer agreement were quantified using the intraclass correlation coefficient (ICC). For binary visual scores, inter-observer agreement was quantified using Cohen’s kappa.

## Results

Agreement for repeated measurements was high. For continuous outcomes, intra- and inter-observer agreement was excellent (coefficients > 0.90), indicating high reproducibility across observers and repeated measurements by the same observer.

In the automatic reconstruction protocol (G1), the software-generated panoramic curve preferentially followed the mandibular arch. The automatically selected panoramic thickness also exceeded the maximum thickness available for manual selection in the software.

At the crown level, axial and reformatted panoramic measurements differed significantly in all three protocols (*p* ≤ 0.05). At the alveolar crest (bone) level, the difference was significant in G1 and G3 protocols. For G2, the bone-level difference was small and not statistically significant (*p* = 0.051) (Table 1).

**Table 1:** Comparison between the measurements of axial cut and reformatted panorama within each group (Paired T-test)

Group	Measurement	Mean	S.D.	Mean diff.	S.D.	95% CI		T	P value
						Lower	Upper		
G1	Crown Axial	8.5650	0.86039	2.51143	0.09146	2.32377	2.80	27.460	0.00*
	Crown Pan	6.0536	0.63507						
	Bone Axial	9.2954	0.94956	1.83107	0.13342	1.55732	0.90	13.725	0.00*
	Bone Pan	7.4643	1.15188						
G2	Crown Axial	8.5654	0.82277	0.87250	0.11344	0.63975	2.00	7.692	0.00*
	Crown Pan	7.6929	0.84368						
	Bone Axial	9.2957	0.95018	− 0.08643	0.04223	− 0.17308	0.57	-2.047	0.051
	Bone Pan	9.3821	0.99705						
G3	Crown Axial	8.5450	0.83929	0.97000	0.13905	0.68470	1.25530	6.976	0.00*
	Crown Pan	7.5750	0.82938						
	Bone Axial	9.3393	0.97992	− 0.08214	0.03675	− 0.15755	− 0.00674	-2.235	0.034*
	Bone Pan	9.4214	1.01228						

*: Significant at *P* ≤ 0.05

Between-group comparisons confirmed that G1 produced significantly larger discrepancies than both G2 and G3 at both crown and bone levels (*p* < 0.001). No significant differences were found between G2 and G3.

A consistent pattern of measurement underestimation was observed across protocols, particularly in G1. Measurement error showed a significant positive correlation with panoramic interslice distance (PISD) at the crown level in all protocols (*r* = 0.555–0.846, *p* < 0.001), indicating that greater deviation from the center of the panoramic curve resulted in increased distortion. At the bone level, this relationship remained strong and significant only in G1 (*r* = 0.836–0.906, *p* < 0.001), while it was weak and non-significant in G2 and G3.

Regarding subjective image quality (Table 2), significant differences were found among protocols (*p* < 0.001). G1 achieved the highest overall scores. However, it showed reduced visibility of the inferior alveolar canal. Manual protocols demonstrated improved inferior alveolar canal visibility but were associated with reduced bone display, reflecting frequent bone cutoff artifacts. Smile line normality differed significantly between groups, with G2 showing the most favorable outcomes. No tooth overlap was observed in any protocol.

**Table 2:** Subjective image quality comparison across reconstruction protocols

Parameter	G1	G2	G3	Overall *p*-value
Smile line	24/28	26/28	13/28	< 0.001*
DEJ visibility	26/28	22/28	22/28	0.258
Absence of distortion	28/28	21/28	17/28	0.002*
Image extension	28/28	23/28	23/28	0.061
Inferior Alveolar Canal visibility	13/28	20/28	20/28	0.084
Bone display	28/28	9/28	8/28	< 0.001*
Teeth overlap	28/28	28/28	28/28	1.000

* Statistically significant (*p* < 0.05).

## Discussion

Images should be assessed for both geometric accuracy and visual interpretability before they are relied upon clinically for diagnosis or treatment planning. Accurate radiographic measurements (geometric/quantitative accuracy) and reliable visual assessment (qualitative accuracy) of dental radiographs are critical before any intervention [12]. Accordingly, this study evaluated both aspects on three CBCT-reformatted panoramic images. To the best of our knowledge, this approach has not been previously reported. CBCT-reformatted panoramic images offer several visual advantages (like conventional panoramic images) by providing a simple, broad overview, in addition to the expectation to reduce certain conventional limitations [13]. To evaluate these features systematically, a novel 7-item visual scoring system was developed.

Regarding quantitative accuracy, CBCT is considered a reliable modality for linear measurements [6, 14]. However, evidence regarding the accuracy of measurements performed specifically on CBCT-reformatted panoramic images remains limited [13]. The present study focused on horizontal measurements at two clinically relevant levels commonly used in implant dentistry and orthodontic assessment [7]. Reliable radiographic measurement requires a consistent definition of points, typically through standardized reference lines and their intersections [5]. This rationale guided the use of overlays and reference lines in the present protocol. To further standardize measurement levels after plane reorientation, 3D volume rendering was used to confirm alignment across protocols, which is another novel protocol in this study.

Measurements on flattened panoramic reconstructions are straight lines, while the actual anatomical path follows the curvilinear arch form (chord vs. arc length). In other words, straight-line measurements systematically underestimate true curvilinear distances, with the magnitude of error proportional to the curvature at that location. This effect is minimal in relatively straight and small segments as the crown of the central incisor, where the distance between mesial and distal tooth surfaces are nearly straight (as can be identified in Fig. 4). The error noticed in this study was mainly due to different reconstruction axes or center of reconstructed panoramic curve (quantified by PISD), which produced different degrees of flattening and geometric discrepancies. Also, the presented methodology reflected currently used clinical workflows, where practitioners make straight-line measurements on flattened panoramic CBCT views.

A recognized contributor to conventional panoramic limitations is the thin focal trough in the anterior region and the sensitivity to head positioning [15, 16]. CBCT-reformatted panoramic views were expected to mitigate some of these limitations because of the ability to define a thicker and more customized panoramic layer. In this context, the software permitted a maximum manual panoramic curve thickness of 25 mm, whereas the automatic function used a larger thickness. This is a relevant software-dependent factor that warrants attention in future updates. Allowing a wider range of manual thickness options might reduce the bone cutoff observed in manually reformatted panoramas. However, increasing thickness may also reduce visibility and accuracy by including additional non-relevant tissues; [17] therefore, improved customization and enhancement options would be needed to balance coverage and displays.

The mean discrepancy was largest in the automatic protocol (approximately 2.5 mm at the crown level and 1.8 mm at the bone level). In the manual groups, crown-level discrepancies were sub-millimetric, and bone-level discrepancies were minimal (less than 0.1 mm). From a statistical perspective, most comparisons showed significant differences except for G2 at the bone level. Three considerations may help interpret these findings considering clinical relevance and other results.

First, the clinical acceptability of measurement error should be considered. Ideally, clinically tolerable radiographic measurement errors should be sub-millimetric [5], although the acceptable threshold depends on the intended intervention [5, 9]. For a voxel size of 0.2 mm, an error of approximately 0.2 mm at each measurement endpoint (approximately 0.4 mm total) has been suggested [18]. Therefore, an average error of around 0.5 mm may be acceptable in some settings despite statistical significance [5, 18].

Second, the insignificant difference between G2 and G3 suggests that limited variability in plane orientation may be tolerated without a clinically meaningful loss of accuracy. Nikneshan et al. concluded that changing plane orientation within − 12° to + 12° affected linear measurement accuracy by less than 0.5 mm and described it as clinically acceptable [19].

Third, the error pattern tended toward underestimation of the mesio-distal dimension, consistent with other studies [18]. This error (absolute and percentage) correlated significantly with PISD, reflecting the distance between the measurement site and the center of the panoramic curve. This may be explained by projection geometry in which structures positioned farther from the curve center are represented with greater distortion, particularly in the presence of anterior inclination. The strong positive correlation observed only in G1 at the bone level may be related to the automatic curve being preferentially aligned with the mandibular arch (effectively increasing the anterior offset for maxillary measurements). This could create greater positional deviations between the bone measurement site and the curve center, thereby amplifying distortion effects. In the manual protocols, standardized curve placement at the alveolar crest level has minimized this positional variability, reducing the influence of PISD on bone measurements. This also highlights the importance of using separate maxillary and mandibular panoramic curves instead of a single average arch when clinically valuable to accommodate the natural discordance between maxillary and mandibular arch forms (particularly in cases with significant arch form differences).

Automatic reconstruction may rely on synthesis approaches in which the mandibular dental curve performs better [20]. Defining the panoramic curve on a single axial level can be limiting because the maxillary and mandibular arches do not coincide in three dimensions, potentially resulting in deficient anatomic coverage or compromised contrast if excessive thickness is used [17, 20]. AI-assisted algorithms may offer opportunities for improvement by fitting the curve not only horizontally but also vertically across anatomical levels [20, 21]. The present findings emphasize the importance of plane orientation and the axial level used during construction. Future software development could also provide vertical control points so that operators can adjust control points in both horizontal and vertical dimensions.

On the other hand, Almeida et al. and Alkhader and Hudieb reported accurate measurements on reformatted panoramic views [13, 22], which contrasts with our findings. This may be due to differences in the measurement location, the software used, and our strict standardization protocol. The debate about the validity of measurements should be considered by clinicians. For example, it is not recommended for orthodontists to depend on reformatted panoramic images for mesio-distal analysis if they do not provide a proven improvement over conventional panoramic imaging in that respect [23]. Also, in orthodontic follow-up, changes in tooth long axis could also affect how teeth appear on reformatted panoramas, as clearly shown in our study.

Improving CBCT-reformatted panoramic views could reduce the perceived need for additional conventional panoramic exposures in patients who already have CBCT datasets available [18, 24]. Therefore, the comparison that includes the points shown in the criteria presented seems timely and needed. This is clinically relevant because user preference for conventional images does not justify additional radiation exposure [25, 26]. Nevertheless, CBCT should not be used when conventional imaging is sufficient for diagnosis, given the higher radiation dose [5, 12, 27]. This study does not advocate replacement of conventional panoramic radiographs by reformatted panoramic views. CBCT examinations generally involve a higher radiation dose than conventional panoramic radiography. Therefore, CBCT should not be prescribed solely for the purpose of obtaining panoramic-like images. However, when a CBCT scan has already been justified for diagnostic reasons, reformatted panoramic images can provide additional information without exposing the patient to radiation from an extra panoramic examination. In such situations, panoramic reconstruction may support the ALADA principle by maximizing information obtained from the already acquired CBCT dataset.

The superior subjective image quality observed in G1 may be related to proprietary vendor-specific enhancement processes integrated into the automatic reconstruction workflow. These processes may include adaptive contrast adjustment, noise reduction, edge enhancement, and optimization of reconstruction thickness. Because the software functions as a proprietary ‘black box’, the exact contribution of each component cannot be determined in detail. Greater transparency from software manufacturers regarding the reconstruction parameters, processing steps, and enhancement algorithms used in automated panoramic reconstruction would be highly beneficial for researchers. Such transparency would improve understanding of the factors influencing image qualitative and quantitative assessment, facilitate independent validation, and support further optimization and standardization of digital workflows.

On the other hand, the automatic enhancement of the reformatted panoramic views appeared to reduce visibility of an important anatomical landmark (as IAC), emphasizing the need to supplement reformatted panoramic interpretation with other CBCT views when IAC localization is clinically important [28]. This supports the distinction between visually appealing images (pretty images) and diagnostically optimal images.

Beyond inferior alveolar canal visibility, significant differences were observed in smile-line appearance, absence of distortion, and bone display. The automatic protocol achieved higher scores for bone display, whereas the nasal spine-oriented manual protocol demonstrated the most favorable smile-line appearance. DEJ visibility, image extension, and tooth overlap showed no statistically significant differences between protocols, suggesting that these image characteristics were relatively stable regardless of reconstruction approach. CBCT-reformatted panoramic images can address some inherent limitations of conventional panoramic imaging by enabling customization of the focal trough (panoramic curve), which could be viewed as part of personalized dental imaging applications [21]. CBCT also allows post-acquisition plane reorientation and position standardization, which may reduce position-related errors compared with conventional panoramic radiography [29]. The ability to integrate panoramic assessment with simultaneous evaluation of cross-sectional and three-dimensional views from the same dataset is another major advantage.

One of the limitations of the present study is the lack of an anatomic reference standard. Physical measurements on dry skulls were not selected because they do not reproduce real clinical imaging conditions and lack soft-tissue attenuation, which may influence image reconstruction and display characteristics [30]. Furthermore, they require special ethical considerations and would not provide the number of cases needed. Since the primary objective was to compare different panoramic reconstruction workflows derived from the same CBCT dataset, axial CBCT measurements were used as reference standard, as previous studies have demonstrated excellent agreement with direct physical measurements [31]. Radiographic reference has been used before in literature supporting this methodological choice [28].

The present study focused only on the anterior maxillary region because this area was expected to be particularly susceptible to reconstruction-related distortion, mainly due to arch curvature and tooth inclination. This study was designed as a proof-of-concept investigation rather than a comprehensive assessment of all anatomical regions. In the same context, only relatively short measurement distances were included in this study. Future investigations should evaluate longer and/or curved distances, such as inter-canine widths or multi-tooth segments, where the effects of panoramic flattening and arch curvature may be more pronounced.

The present findings were obtained using a single CBCT software platform. Since panoramic reconstruction algorithms, enhancement filters, automation methods, and curve-generation tools differ among software packages, direct extrapolation of the present results to other platforms should be undertaken with caution. Furthermore, multicenter investigations incorporating different devices, software packages, and reconstruction settings, different dental regions, variable distances and curvatures, and other clinically relevant measurement types are recommended to improve generalizability and establish standardized reconstruction guidelines.

## Conclusion

The primary clinical value of the reformatted panoramic image is to provide a comprehensive overview including rapid visualization of anatomical relationships, preliminary assessment of vital structures, facilitate localization of pathological entities, and help in effective patient communication. However, they should not be relied upon as a source for precise linear measurements. Clinicians should perform and verify critical measurements using axial, cross-sectional, and other multiplanar views of the CBCT data, particularly when treatment decisions involve implant placement, orthodontic space analysis, and any clinical procedures requiring sensitive measurements. Manual reconstruction protocols may improve measurement accuracy within the inherent limitations compared with fully automated reconstruction because of the considered anatomy and orientation.

## Data Availability

Data to support the findings of this study are available upon reasonable request from the corresponding author.
